# Progress in Ophthalmology

**Published:** 1899-10-14

**Authors:** 


### PROGRESS IN OPHTHALMOLOGY.

**Treatment of Trachoma.**—Trachoma seems to be such an intractable disease as to call for many remedies. Like its sequela entropium, the number of methods of cure is an indication of their success. The latest method seems to be that of Keiper,[1] of Indiana, who mentioned a number of experiments performed to prove that substances could be made to penetrate the tissues by means of the electric current. He proposes to apply this in the treatment of trachoma. The method suggested was based on the following experiment. Take a carbon electrode attached to the negative pole of a battery, and a pure copper needle attached to the positive pole, and introducing the needle into a piece of meat turn on the current. On cutting the meat open the region around the needle will be found of a green colour, and a chemical examination shows this to be due to the copper oxychloride which has been formed. As this substance has a decidedly germicidal action, it is relied upon to destroy the trachoma granules when the copper pole is applied to the conjunctiva. In the discussion on this subject, Huitzinga considered that if we believed trachoma to be the result of a specific infection, then this treatment deserved more than a passing notice. He held that such a current would penetrate the conjunctiva, and destroy or neutralise the cause of trachoma. He said that the distance between the anode and the cathode should never be less than five inches, as the amount of chlorin ions between the poles must be sufficient to meet any demands that might be made on them.

**The Abortive Treatment of Gonorrhœal Ophthalmia.**—H. D. Jamison[2] recommends for the abortive treatment of gonorrhœal ophthalmia the following method. Cleanse the eye thoroughly with a solution of boracic acid, take a strong pair of scissors, preferably straight with blunt points, placing one blade beneath the palpebral conjunctiva at the outer canthus, make a cut directly outwards, through skin and conjunctiva. Then, grasping the upper lid with the other hand, pull it forwards and upwards; in doing this you put the tarsal ligament on the stretch, and it can readily be found between the skin and the palpebral conjunctiva. This is cut by a smaller pair of scissors, and then the upper lid becomes loose, and one is enabled to expose thoroughly the upper conjunctival cul-de-sac and so to lessen the pressure on the lymphatics supplying the cornea. Should there be much swelling and chemosis following the application he does not think it necessary to stitch the edges of the wound together, as only a temporary effect from the operation is desired.

The application of nitrate of silver, gr. xl—ʒ, should be made with a small pledget of cotton, the operator being careful not to touch the cornea. Immediately neutralise the silver with a sodium chloride solution, then begin the application of iced cloths, changing them every ten seconds till the reaction has subsided; in the meanwhile the eye is kept clean with a saturated solution of boracic acid. It may be necessary to make a second application of the silver nitrate in twenty-four hours, but he says in the case of several patients so treated he did not find it necessary. There was but a slight discharge on the second day, and the reaction rapidly subsided under the vigorous use of iced cloths. The small operation on the external canthus is done with but slight pain to the patient by the subcutaneous injection of a 4 per cent solution of cocaine; and, as the result of this treatment depends entirely on the thorough application of the silver nitrate, he thinks the preliminary operation very essential, as it enables us thoroughly to expose the conjunctiva, which is otherwise impossible.

Dr. Mary R. Wilson[3] quotes rather a peculiar case where what might be called a tremendous hæmorrhage followed the section of an internal rectus, a most unusual occurrence. She certainly mentions that the child was very refractory, and that the tenotomy was done with great difficulty. The operation was done under cocaine, and she says the moment the operation appeared to be completed a jet of what seemed to be arterial blood issued from the conjunctival wound, and in the course of a few seconds the eyeball was thrust forward, and the lids so greatly thickened by effusion of blood into their tissues that removal of the speculum was a difficult matter. A pad and roller bandage were firmly applied, and the child put to bed. Two and a half hours afterwards the eye was examined, when the lids were found to be much distended. There had been some pain, which it was said had disappeared. The next day the lids were so swollen they could not be opened, while the swelling had extended to the side of the face; there was no pain. The temperature rose to 99 deg. Fahr. The eye was kept firmly bandaged. Two days later temperature was again normal, the discoloration and swelling of lids continued, but they were slightly opened; the cornea was bright. The eyeball was still proptosed, but not markedly, the movements of the eye were good. Next day the eye was better in every way, but the ecchymosis had extended to the other eye. A fortnight later a faint discoloration still persisted in lids and cheek; there was also bright red ecchymosis of the ocular conjunctiva; the amount of squint was about the same as before operation. Eventually under ether the internal rectus was again divided, and external rectus advanced; this had the desired effect of straightening the eye, and this time there were no untoward results.

**Epiphora.**—Dr. J. H. Woodward,[4] in summing up his paper as to the treatment of epiphora, says: The management of epiphora evidently consisted in something more than simply probing the nasal duct. The removal of a V-shaped segment from the posterior wall of the punctum and canaliculus was the most satisfactory procedure. Having done this, the patency of the drainage system could be determined by injecting boric acid through the canaliculus with the aid of a hypodermic needle. It was well to avoid probing whenever possible. The simple slitting of the canaliculus was not sufficient. For a number of years he had been accustomed to remove a portion of the posterior wall of the canaliculus, and was convinced of the effectiveness of this method. Probes should be used in these cases on the same principles that govern the introduction of sounds into the urethra. Syringing the tear sac might be practised daily until the inflammation of this sac has been subdued. Lachrymal abscesses should be treated like other abscesses. In bad cases the surgeon might be tempted to excise the lachrymal sac, but in such instances this should not be done until the effect of curetting the sac had been tried. Even extirpation of the entire lachrymal gland, it should be remembered would not cure some cases of epiphora. The removal of the gland appeared to be a perfectly safe operation, but this procedure was very seldom required. Cocaine would produce sufficient anæsthesia for nearly all these cases.

[1] Brit. Med. Jour., July 22, 1899. [2] Med. Rec., June 10, 1899. [3] Lancet, July 15, 1899. [4] Med. Record, June 10, 1899.

